# The Role of Cerebral Metabolism in Improving Time Pressured Decisions

**DOI:** 10.3389/fpsyg.2021.690198

**Published:** 2021-07-20

**Authors:** An Thanh Vu, David A. Feinberg

**Affiliations:** ^1^San Francisco VA Health Care System, San Francisco, CA, United States; ^2^Department of Radiology and Biomedical Imaging, University of California, San Francisco, San Francisco, CA, United States; ^3^Advanced Magnetic Resonance Imaging (MRI) Technologies, Sebastopol, CA, United States; ^4^Helen Wills Neuroscience Institute, University of California, Berkeley, Berkeley, CA, United States

**Keywords:** fMRI, accelerated, time pressure, decision making, initial dip

## Abstract

Speed-accuracy tradeoff (SAT) theory dictates that decisions can be made more quickly by sacrificing accuracy. Here we investigate whether the human brain can operate in a brief metabolic overdrive to overcome SAT and successfully make decisions requiring both high levels of speed and accuracy. In the context of BOLD fMRI we expect “a brief metabolic overdrive” to involve an increase in cerebral oxygen metabolism prior to increased cerebral blood flow–a phenomenon known as the “initial dip” which results from a sudden drop in oxyhemoglobin in perfusing blood. Human subjects performed a motion discrimination task consisting of different difficulties while emphasizing either accuracy (i.e., without time pressure) or both speed and accuracy (i.e., with time pressure). Using simultaneous multi-slice fMRI, for very fast (333 ms) measurement of whole brain BOLD activity, revealed two modes of physiological overdrive responses when subjects emphasized both speed and accuracy. The majority of subjects exhibited the hypothesized enhancement of initial dip amplitude in posterior visual cortex (PVC) with the size of the enhancement significantly correlated with improvement in behavioral performance. For these subjects, the traditionally analyzed post-stimulus overshoot was not affected by task emphasis. These results demonstrate the complexity and variability of the BOLD hemodynamic response. The discovered relationships between BOLD response and behavior were only observed when subjects emphasized both speed and accuracy in more difficult trials suggesting that the brain can perform in a state of metabolic overdrive with enhanced neural processing of sensory information specifically in challenging situations.

## Introduction

**Figure 1:**
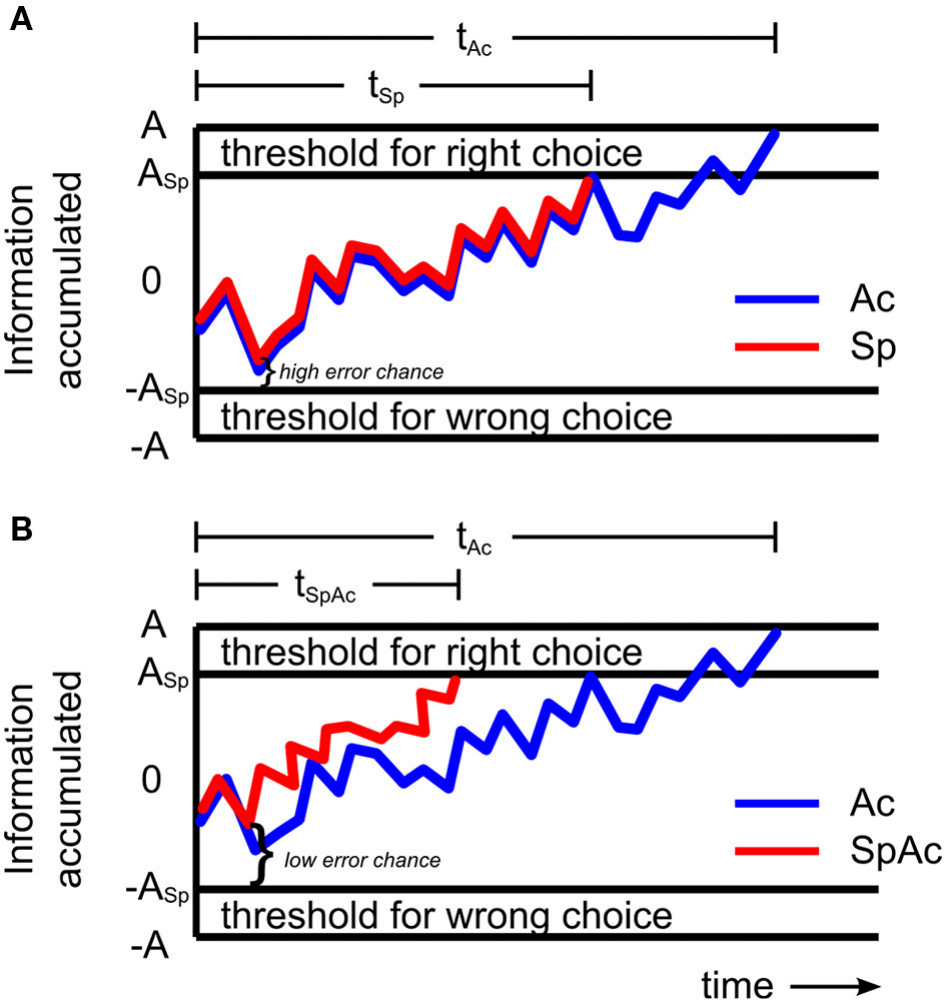
Models of sensory information accumulation during a left-right motion discrimination task. **(A)** Model with constant drift rate. The plotted graph shows the amount of information a subject may accumulate over time under accuracy (blue line) and speed (red line) emphasis. The subject responds once the accumulated information reaches the response threshold. When only accuracy is emphasized, response threshold A is relatively high resulting in relatively long response time t_Ac_. When only speed is emphasized, the rate of information accumulated (drift rate) remains the same but response threshold, A_Sp_, is relatively lower resulting in a shorter response time (t_Sp_ < t_Ac_) and higher chance for error. **(B)** Model with increased drift rate. When both speed and accuracy are emphasized, response threshold, A_Sp_, may be relatively low, while drift rate becomes relatively fast (red line) resulting in shorter response times (t_SpAc_ < t_Ac_) and lower chance for error. By increasing drift rate sufficiently to overcome accuracy losses which would have been caused by a reduction in response threshold, subjects can make decisions with high levels of both speed and accuracy.

When encountering debris on a crowded freeway or falling rocks on a mountain road, the decision to break or swerve could be the difference between life and death. Such critical decisions must be made with both speed and accuracy. However, prior behavioral studies on the speed-accuracy tradeoff (SAT) suggest that these critical decisions are likely to fail—since decisions cannot be both fast and accurate [i.e., accuracy must be traded for speed (Wickelgren, 1977; Forstmann et al., 2008; van Veen et al., 2008; Bogacz et al., 2010)]. The traditional model of SAT holds that for a given stimulus, subjects accumulate sensory information at a constant drift rate (Ratcliff, 1978, 2002; Wagenmakers et al., 2007). Therefore, decisions that are made more quickly (by reducing response threshold; Figure 1A) will be made with less information and thus less accurately.

However, more recent studies (Heitz and Schall, 2012; Ho et al., 2012) revealed that the mechanisms behind SAT can involve changes in not only response threshold but also drift rate. Importantly, Ho et al. (2012) found that drift rate can actually increase when accuracy was emphasized over speed. This increase was correlated with BOLD activity in primary visual cortex suggesting that subjects improved their drift rate (and thus accuracy) by enhancing the processing of visual sensory information. Heitz and Schall (2012) found similar results in the spike trains of visually responsive frontal eye field neurons of the rhesus monkey with the additional key finding that drift rate can increase independently of decreasing response thresholds—suggesting the existence of a behavioral mechanism for overcoming SAT. Unfortunately however, both studies were ultimately consistent with SAT theory, reporting that the net changes in drift rate and response threshold resulted in either slower, more accurate decisions or faster, less accurate ones. In order to overcome SAT and have decisions that are both fast and accurate, drift rate must increase sufficiently to overcome any accuracy loss that would be due to a reduction in response threshold. By increasing drift rate independent of stimulus input (e.g., by enhancing the neuronal processing of sensory information), subjects could potentially overcome SAT and successfully respond to situations requiring high levels of both speed and accuracy (Figure 1B). From the results of Ho et al. (2012) and Heitz and Schall (2012), it is expected that such an improvement in processing of visual information will require rapid onset of relatively intense neural activity.

Regions of the brain processing information generally increase in cerebral metabolic rate of oxygen (CMRO-_2_) while maintaining a net surplus of oxygen supply through overcompensation of cerebral blood flow (CBF) (Fox and Raichle, 1986; Fox et al., 1988). This hyperemic response results in the ~6 s post stimulus positive overshoot response commonly used as a neural correlate in BOLD fMRI (Logothetis and Wandell, 2004). However, rapid onset of relatively intense neural activity prior to increases in CBF can result in net deficits in oxygen supply relative to baseline (Ernst and Hennig, 1994; Hennig et al., 1995; Hu et al., 1997; Thompson et al., 2003; Ances, 2004; Takano et al., 2006; Vanzetta and Grinvald, 2008; Hu and Yacoub, 2012)–e.g., a state of metabolic overdrive. Prior studies combining hemodynamic and electro-physiological recordings suggest that brief periods of intense neural activity result in an early (2–3 s post stimulus onset) negative BOLD response often referred to as the initial dip (Thompson et al., 2003; Nemoto et al., 2004). This is distinct from the sustained negative BOLD response indicative of suppressed neural activity (Shmuel et al., 2006).

The initial dip was first reported in optical imaging studies as an initial decrease in oxy-hemoglobin and increase in deoxy-hemoglobin concentration (Grinvald et al., 1991; Malonek and Grinvald, 1996). Subsequent studies suggested that the biophysical mechanism behind the initial dip is an increase in CMRO_2_ prior to an apparent vascular overcompensation of increased CBF (Ernst and Hennig, 1994; Hennig et al., 1995; Hu et al., 1997; Thompson et al., 2003; Ances, 2004; Takano et al., 2006; Vanzetta and Grinvald, 2008; Hu and Yacoub, 2012). Others have suggested that an initial increase in cerebral blood volume (CBV), due to vessel dilation, is also responsible for the initial dip (Nemoto et al., 2004; Sirotin et al., 2009; Chen et al., 2011). In BOLD fMRI, these dynamic changes in CMRO_2_, CBF and CBV impact deoxy-hemoglobin concentration and hence magnetic susceptibility dephasing and signal decay. The relationship between these biophysical properties and BOLD signal change relative to baseline can be intuitively described by the Davis equation (Davis et al., 1998; Buxton, 2013) as:

(1) $$\begin{array}{l} \frac{\Delta S\left(t\right)}{S_{0}}=M\left[1-v\left(t\right){\left(\frac{r\left(t\right)}{f\left(t\right)}\right)}^{\beta }\right] \end{array}$$

where M is a constant depending on magnetic field strength, echo time (TE), as well as baseline physiological parameters; *v, f* and *r* represent CBV, CBF, and CMRO_2_, respectively, normalized relative baseline; and β represents the effect of diffusion on the signal due to venous susceptibility gradients.

Although the initial dip is not utilized in cognitive neuroscience studies due to its elusive nature (Ances, 2004; Vanzetta and Grinvald, 2008; Hu and Yacoub, 2012), prior studies have found it to be more spatially specific (Menon et al., 1995; Nemoto et al., 2004; Sirotin et al., 2009; Chen et al., 2011) as well as more linearly correlated (Duong et al., 2000; Thompson et al., 2003; Nemoto et al., 2004) to neural activity relative to the larger positive overshoot response. In these latter studies, strong stimulation resulted in saturated positive overshoot responses while the initial dip remained highly correlated with stimulus condition. For these reasons we sought out the initial dip as a likely biomarker of the metabolic overdrive used when making fast, accurate decisions.

The aim of this article is to explore whether subjects can indeed overcome SAT to achieve both speed and accuracy in task performance and test whether the BOLD initial dip is a viable biomarker of a metabolic overdrive. To accomplish this, we instructed human subjects to perform a motion discrimination task while emphasizing either accuracy or both speed and accuracy. Fitting a mathematical model to the behavioral data revealed that subjects were able to improve both speed and accuracy by increasing drift rate and reducing response thresholds. Using recent advances in fMRI to reduce whole brain scan time from 3,000 ms down to 333 ms (Feinberg et al., 2010; Moeller et al., 2010; Setsompop et al., 2012; Feinberg and Setsompop, 2013), we were able to make precise measurements of the initial dip, shed light on why it has been so elusive, and reveal its involvement in overcoming SAT.

## Methods

### Behavioral Task

Fifteen subjects (seven male, ages 21–53) provided informed consent and participated in both Ac and SpAc emphasized runs. All experimental protocols were approved by the Committee for the Protection for Human Subjects at the University of California, Berkeley. Subjects performed the motion discrimination task while lying in the fMRI scanner and used their preferred hand to hold a button box to indicate their decision (left or right).

**Figure 2:**
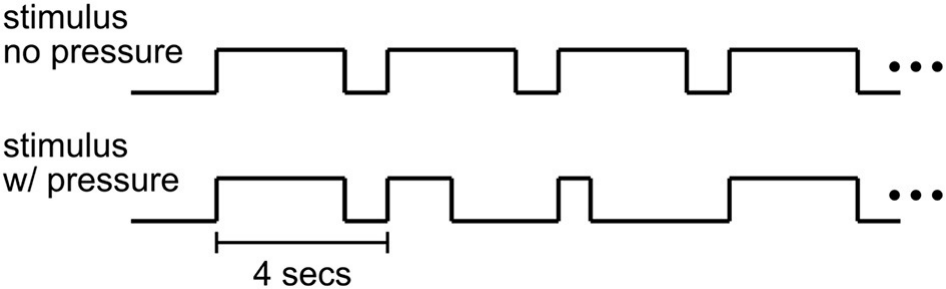
Stimulus timecourse. In Ac emphasized (no pressure) runs, subjects could respond anytime within a 3 s period of stimulus presentation. In SpAc (pressure) runs, half of the trials required a response within a shorter stimulus duration (between 400 and 1,000 ms).

The time course of a trial in the decision-making experiment is shown in Figure 2. Each trial of the Ac runs began with 3 s of dynamic random-dot stimulus at the center of the screen within a circular aperture of radius 6 degrees. In each trial, the direction of motion was randomly chosen to be leftward or rightward. The stimulus density was 1.77 dots/deg^2^ with individual dots moving at a speed of 5 deg/s. All dots were assigned a random initial position, direction and duration of motion. Each dot was presented for 12 frames (200 ms) and was then re-assigned a random position. The probability that a dot would be assigned the chosen direction of motion (percent motion coherence) determined the task difficulty. For each trial, the motion coherence value was selected randomly from the set (0, 3.2, 6.4, 12.8, 25.6, 51.2%; mapped to task difficulty values 6, 5, 4, 3, 2, and 1 respectively) such that 12 repetitions of each percent coherence were presented per run. Trials with difficulty value 6 (0% coherence) were omitted from behavioral analysis since no actual correct answer existed for these trials resulting in some subjects consistently making no decision within the 3 second time limit. To estimate the fMRI baseline, every 24th stimulus trial was followed by a blank trial (fixation only) and every run ended with 10 s of fixation.

Subjects were instructed to judge the direction of the moving random dots as quickly and as accurately as they could. Subjects were required to make their decision during the period of the motion stimulus (3 s) or the stimulus would be extinguished automatically. Prior to the start of the subsequent trial (4 s inter-trial-interval), subjects were provided with visual feedback regarding whether they had made the correct choice, incorrect choice, or failed to make a choice in time via fixation color change. Subjects were instructed to maintain fixation on a small square at the center of the screen.

For SpAc runs, half of the trials had a stimulus duration that was selected randomly from the set (400, 700, 1,000 ms). The shortest duration of 400 ms was chosen to be at or below subjects' fastest response times to ensure subjects perceived the SpAc emphasis. Each run consisted of 72 trials. Subjects performed an initial training session of at least one Ac and one SpAc run followed by the actual experiment of at least four Ac and SpAc runs in counter balanced order. Subjects were told prior to each run whether the run would be Ac or SpAc. Runs were counter balanced such that each combination of coherence and task condition (Ac or SpAc) was presented 36 times. The behavioral effects of SpAc were obtained only from data of full length (3 s) trials.

### MRI Parameters

Functional scans were conducted using a 3 Tesla Siemens MAGNETOM Trio scanner with a 32 channel receive coil. Scans were obtained using T2^*^-weighted multiband factor 8, ramp sampling on, echo spacing 0.54 ms, TR 333 ms, TE 35 ms, flip angle 40°, voxel size 3.0 × 3.0 × 3 mm^3^, and FOV 240 × 240 mm^2^. The slice prescription covered 40 axial slices providing whole brain coverage. Each run was 310 s (930 TRs per run).

### Data Pre-processing

fMRI data were pre-processed as described in earlier publications (Kay et al., 2008; Naselaris et al., 2009). Motion correction was performed using FSL (http://fsl.fmrib.ox.ac.uk), supplemented by additional custom Matlab (The Mathworks, Natick, MA) algorithms. For each 310 s run and each individual voxel, drift was modeled by zero, first, second, and third-degree nuisance polynomials. The time-event separable model (Kay et al., 2008) was used to fit 16 s long hemodynamic response functions (HRFs) for each voxel under each motion coherence, stimulus duration, and time-pressure condition. For improved statistical power, a basis-restricted model (discrete cosine transform with degrees 0 through 12) was used instead of the traditional finite impulse response basis. Importantly, the design matrix of the original time separable model was slightly modified in this study to account for trial specific stimulus durations (response times). This was achieved by replicating the shifted binary sequence a number of time points corresponding to the response time divided by TR. Durations less than the TR were scaled proportionally (see Supplementary Figure 3).

### Data Analysis

Mean response times and percent correct target selection were computed for each motion coherence and task condition (Ac and SpAc). Response time was calculated using only correct trials. Importantly, only trials with full stimulus duration (3 s) were included in the analysis. The mean and variance of response times as well as percent correct were fed into the EZ-diffusion model (Wagenmakers et al., 2007) to generate drift rate and response thresholds values per subject, task difficulty, and task condition. The EZ-diffusion model was chosen for its closed form, computationally efficient method to calculate drift rate and response thresholds. Recent studies have demonstrated it to give qualitatively identical results as more complicated, iterative methods (Wagenmakers et al., 2007; Forstmann et al., 2008) especially when response times follow a Gaussian distribution, as was the case in this study. Omission of outlier response times (5–10% most extreme) resulted in almost identical results.

ROIs were selected based on GLM explained variance (*P* < 0.01, Bonferroni corrected for multiple comparisons) and anatomical location based on the Harvard-Oxford cortical atlas supplied by FSL (http://fsl.fmrib.ox.ac.uk). The atlas was registered to each individual subjects' native fMRI space via 12 DOF affine transformation between MNI T1 standard and each subjects' mean fMRI volume. Voxels in posterior visual cortex (PVC) and medial prefrontal cortex (mPFC) were defined as voxels above the explained variance criterion within the “Occipital Pole” and [“Frontal Pole” + “Frontal Medial Cortex”] Harvard-Oxford labeled regions, respectively.

The response strength of the initial dip and positive overshoot was calculated by averaging the TRs corresponding to the 2–3 s and 5.5–6.5 s after stimulus onset, respectively. The one second windows were chosen based on prior studies on the initial dip (Hu et al., 1997). Paired *t*-tests across subjects of initial dip and positive response strengths under Ac and SpAc emphasis were calculated using the mean response strengths across all voxel in each subject's ROI. The sustained suppression found in mPFC was quantified by taking the mean of the BOLD response time course. Paired *t*-tests were calculated similarly using these mean response values.

Significance of the correlation between change in initial dip amplitude and improvement in drift-rate (SpAc–Ac) was determined via permutation testing. This procedure involved randomly assigning drift rate improvements from difficulty levels 4 and 5 of individual subjects to changes in initial dip amplitude of other subjects (without replacement). Subsequently, two-cluster Gaussian mixture modeling was performed. The process was repeated 5,000 times from which the *P*-value was determined as the frequency of observing clusters as large and correlations as strong as those observed.

## Results

We examined the effects of emphasizing accuracy or both speed and accuracy on behavioral performance (response time and accuracy) as well as on the BOLD signal using sub-second scanning of the whole brain with simultaneous multi-slice EPI (Feinberg et al., 2010; Moeller et al., 2010; Setsompop et al., 2012). Similar to previous studies on time-pressured decision making (Forstmann et al., 2008; Bogacz et al., 2010), we asked our subjects to perform a left-right motion discrimination task with various difficulties (motion coherences). However, rather than instruct subjects to focus on either accuracy or speed [which accentuates SAT effects (Wickelgren, 1977; Ho et al., 2012)], we imposed stimulus deadlines to have subjects focus on either accuracy (Ac) or both speed and accuracy (SpAc). Each trial of the Ac runs began with 3 s of dynamic random-dot stimulus during which subjects were allowed to respond. This was followed by a 1 s fixation period. Just prior to the beginning of the subsequent trial, subjects received feedback via a brief color change of the fixation square (green–correct, yellow–no response/too slow, red–incorrect). SpAc runs were the same as Ac runs except that in half of the trials stimulus duration (and thus the time subjects were allowed to respond) was selected randomly from a set of shorter durations (400, 700, 1,000 ms; Figure 2). Importantly, unless otherwise noted, only full (3 s) trials in both Ac and SpAc conditions were used in calculating behavioral performance.

**Figure 3:**
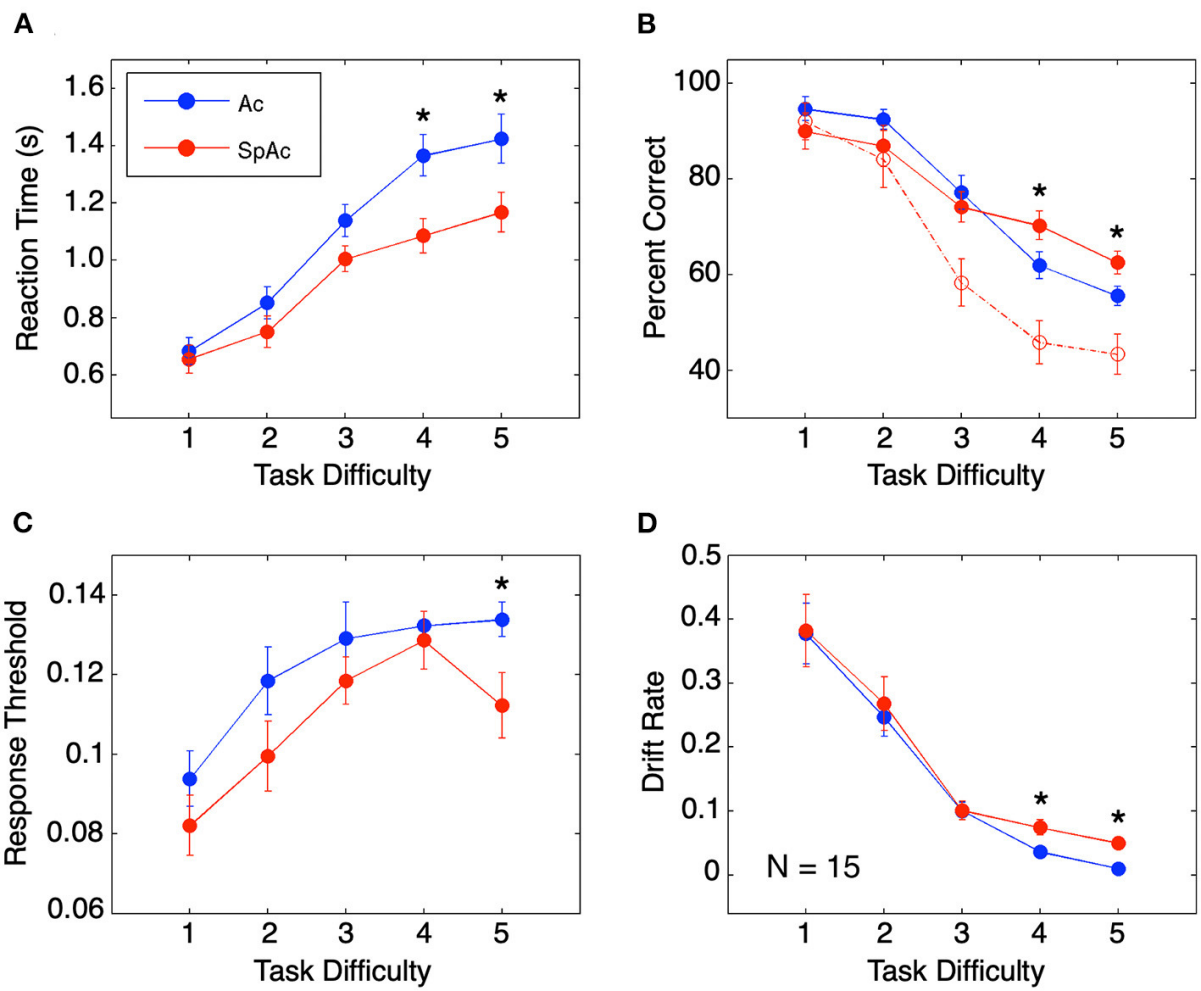
Behavioral performance and model fit. **(A)** Behavioral response time. Response times are longer for higher task difficulties but shorter with SpAc emphasis (*P* < 0.01). **(B)** Behavioral accuracy measured by percent correct. Accuracy declined with increasing task difficulty. On trials of difficulty levels 4 and 5, when subjects had the full 3 seconds of stimuli, accuracy improved with SpAc emphasis (solid red) relative to with Ac emphasis (blue) (*P* < 0.01). Including trials with shorter stimulus durations resulted in reduction of accuracy with SpAc emphasis (dashed red) as reported in prior studies. **(C)** Response threshold. Response thresholds increased with task difficulty but were lower with SpAc emphasis, (*P* < 0.05). **(D)** Drift rate. Drift rate decreased with task difficulty, but increased with SpAc emphasis for difficulty levels 4 and 5 (*P* < 0.01). Error bars are SEM across subjects. Asterisks indicate which SpAc–Ac pairwise comparisons are statistically significant (*P* < 0.05).

Behavioral performance across task difficulties replicated previously reported findings. Relative to higher difficulty trials, lower difficulty trials resulted in both faster and more accurate subject responses (Figures 3A,B). In line with previous SAT studies (Wickelgren, 1977; Forstmann et al., 2008; van Veen et al., 2008; Bogacz et al., 2010), SpAc emphasis (red; Figure 3A) resulted in significantly faster response times than Ac emphasis [blue, *P* < 0.01, paired *t*-test (Hennig et al., 1995)]. Furthermore, when short duration trials were included in accuracy calculation (as was done in prior SAT studies), SpAc emphasis resulted in poorer response accuracy (dashed red vs. blue; Figure 3B). However, by including short duration trials in accuracy calculation, accuracy is biased against SpAc since subjects have as little as 1/7th the time to respond relative to full length, 3 s trials. Importantly, when only full duration trials were included in accuracy calculation, SpAc emphasis significantly improved response accuracy for the two most difficult conditions (solid red vs. blue; *P* < 0.01). These results demonstrate that subjects can improve speed without sacrificing accuracy.

Fitting individual response time and accuracy data to a mathematical model of cognitive decision-making (Ratcliff, 1978, 2002; Wagenmakers et al., 2007) revealed the behavioral mechanisms behind subjects' overcoming of SAT. In line with previous SAT studies (Wickelgren, 1977; Forstmann et al., 2008; van Veen et al., 2008; Bogacz et al., 2010), response thresholds were lower with SpAc emphasis (Figure 3C, *P* < 0.05). Furthermore, drift rates decreased as task difficulty increased (Figure 3D). Importantly, we found that SpAc emphasis resulted in higher drift rates than Ac emphasis for the two most difficult conditions (*P* < 0.01). These results demonstrate the behavioral mechanism for improving response time without sacrificing accuracy is the increase of drift rate.

**Figure 4:**
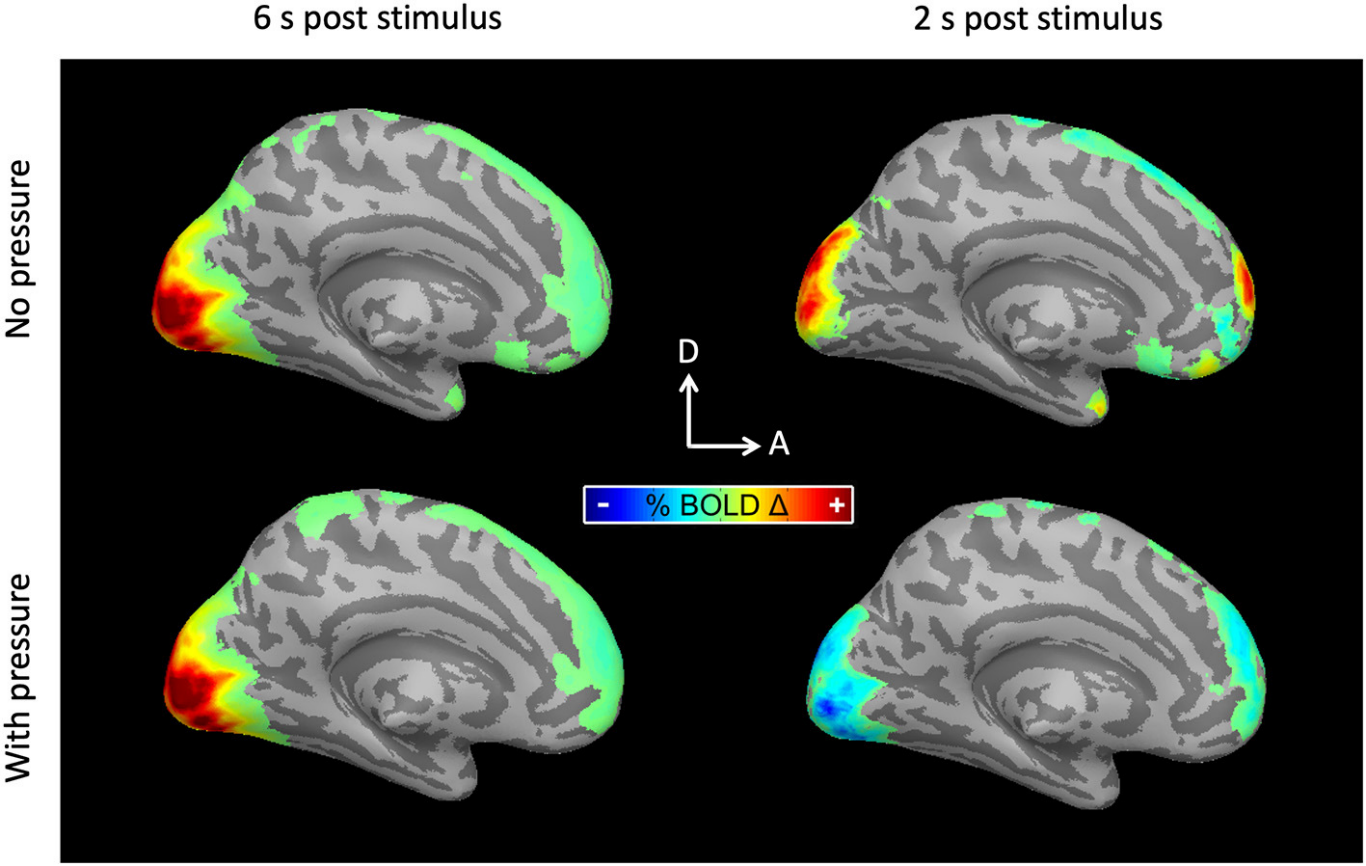
BOLD activations for the majority group average. **(Left)** 6 s post stimulus onset. PVC was activated (responded positively) to moving dot stimuli. The activation was similar for both Ac and SpAc emphasis. **(Right)** 2 s post stimulus onset. Both PVC initial dip activity and mPFC suppression were enhanced with SpAc emphasis.

Taking advantage of the high temporal resolution fMRI used in this study (TR = 333 ms), hemodynamic response functions were measured with Ac and with SpAc emphasis. As previously reported (Singh and Fawcett, 2008), the motion discrimination task evoked strong positive activations in posterior visual cortex (PVC) while suppressing medial pre-frontal cortex (mPFC). Figure 4 shows the average BOLD activation at ~6 as well as ~2 s post stimulus onset for the majority group average (*N* = 10, details below). Qualitatively, the traditionally analyzed positive overshoot did not differ between Ac and SpAc emphasis. However, the difference between task conditions during the initial dip period was striking in both the PCV and mPFC.

**Figure 5:**
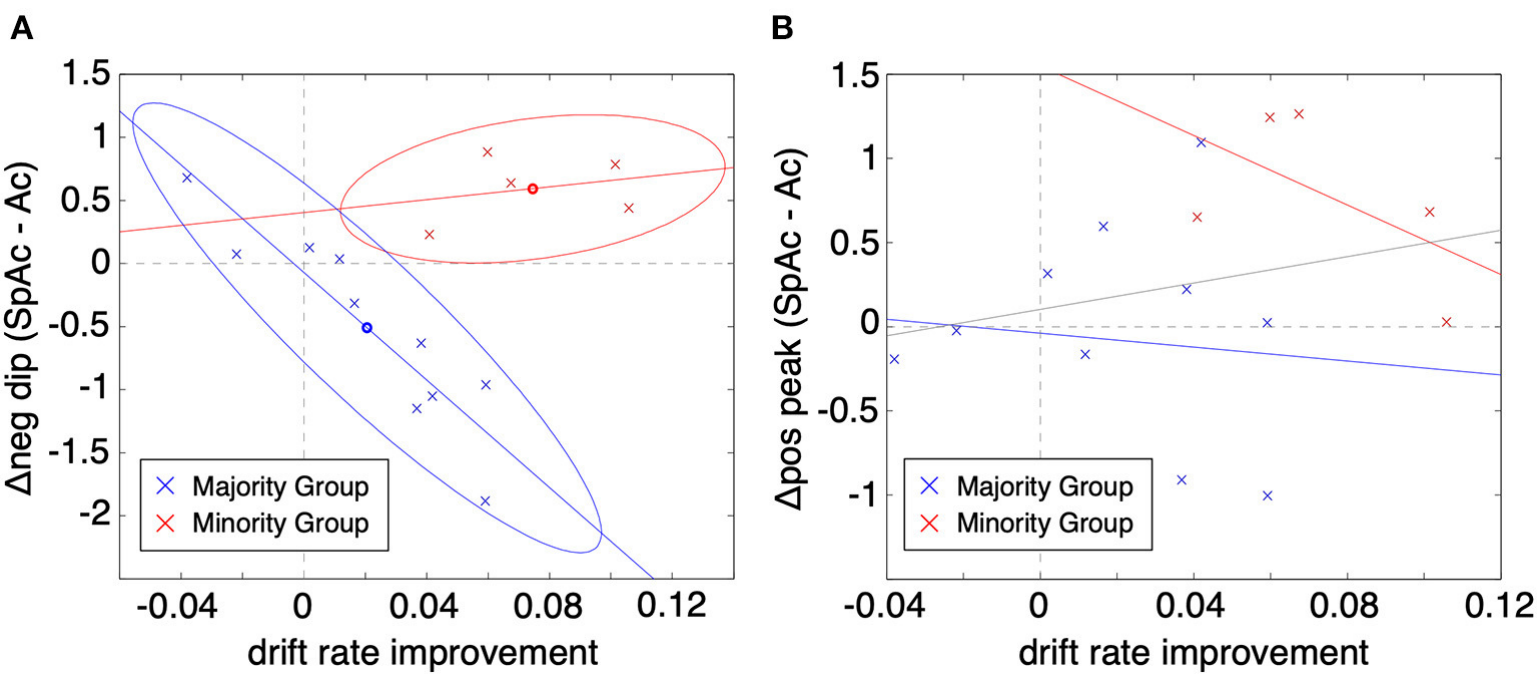
Change in PVC BOLD response vs. improvement in drift rate (SpAc-Ac). **(A)** BOLD initial dip vs. drift rate improvement. Each data point represents a single subject and task difficulty (difficulty values 4 and 5 plotted). Two-cluster Gaussian mixture modeling revealed two distinct classes of responses. The majority group (10 subjects; blue x's) exhibited significant correlation between change in PVC initial dip amplitude and drift rate improvement (*cc* = −0.74, *P* < 0.01 determined via permutation testing). The minority group (red x's) exhibited significantly higher SpAc-Ac drift rate improvements [*P* < 0.01, *t*-test (Ernst and Hennig, 1994)]. **(B)** BOLD positive overshoot vs. drift rate improvement. Neither group had significant correlations between the subsequent PVC positive response (~6 s post stimulus onset) and drift rate improvement (though the correlation of the minority group was marginally significant cc = −0.62, *P* = 0.06).

To test whether the BOLD initial dip is a viable biomarker of metabolic overdrive, we plotted the change in PVC initial dip amplitude (SpAc minus Ac at ~2 s post stimulus onset) vs. the improvement in drift rate (across all 15 subjects averaged over task difficulties 4 & 5; Figure 5A). Given the elusive nature of the initial dip (Malonek and Grinvald, 1996; Nemoto et al., 2004; Sirotin et al., 2009) and the potential for systematic differences between how subjects respond physiologically (Aguirre et al., 1998; Laurienti et al., 2002; Handwerker et al., 2004), we applied Gaussian mixture modeling (Bishop, 2006) to the plotted data which revealed two distinct groups of subjects. The majority group (10 subjects; blue x's) exhibited significant correlation between change in PVC initial dip amplitude and drift rate improvement (*cc* = −0.74, *P* < 0.01, determined via permutation testing; see Methods). In comparison to the majority group, the minority group (red x's) excelled under time pressure with both significantly higher SpAc-Ac drift rate improvements [*P* < 0.01, *t*-test (Ernst and Hennig, 1994)] as well as absolute SpAc drift rates [*P* < 0.05, *t*-test (Ernst and Hennig, 1994)]. Further demonstrating the utility of the initial dip as a biomarker for metabolic overdrive, neither group alone (nor as a single aggregated group) had significant correlations between the subsequent PVC positive response (~6 s post stimulus onset) and drift rate improvement (Figure 5B; though the correlation of the minority group was marginally significant cc = −0.62, *P* = 0.06).

**Figure 6:**
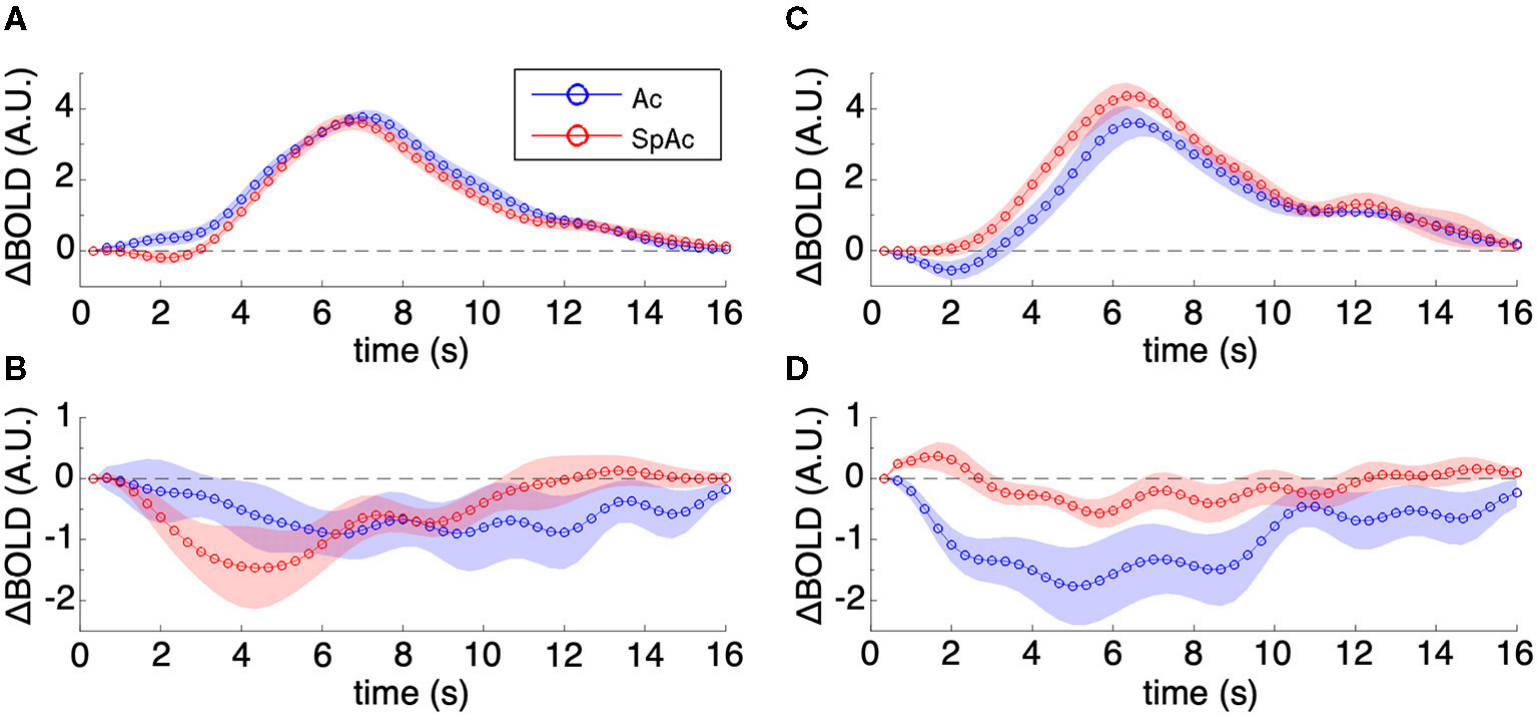
Group mean HRFs in PVC and mPFC. **(A)** Majority group mean HRFs of PVC. SpAc emphasis enhanced initial dip amplitude (*P* < 0.01) while the remainder of the HRF did not change significantly. **(B)** Majority group mean HRFs of mPFC. The HRFs in pre-frontal cortex showed a sustained suppression (*P* < 0.01), which was initially enhanced and then later reduced by SpAc emphasis. **(C)** Minority group mean HRFs of PVC. SpAc emphasis significantly reduced initial dip amplitude and increased the positive overshoot response in the (*P* < 0.05). **(D)** Minority group mean HRFs of mPFC. SpAc emphasis introduced an initial positive dip (*P* < 0.05) followed by milder sustained suppression (*P* < 0.01). Error bars are SEM across subjects.

Visualizing the majority group mean HRFs within PVC (Figure 6A; error bars are SEM across subjects) showed increase in initial dip amplitude (*P* < 0.01, determined via permutation testing) with SpAc emphasis while the remainder of the HRF did not change significantly. The HRFs in pre-frontal cortex showed a sustained suppression [*P* < 0.01, paired *t*-test (Heitz and Schall, 2012)], which was initially enhanced and then later reduced by SpAc emphasis (Figure 6B). This is consistent with initial enhanced suppression of mind wandering in more demanding tasks (Gusnard et al., 2001; Raichle et al., 2001; Mason et al., 2007; Singh and Fawcett, 2008). We also found suppression of the default mode network (e.g., retrosplenial cortex, posterior cingulate, lateral parietal cortex, and mPFC) during our motion discrimination tasks. However, only mPFC showed significant enhancement of this suppression under SpAc emphasis suggesting that time pressure reduces self referential thoughts (Gusnard et al., 2001).

In contrast, the minority group already had a strong initial dip when emphasizing accuracy alone. When these subjects emphasized both speed and accuracy, their BOLD response in PVC exhibited a significant decrease in initial dip amplitude [*P* < 0.001, determined via permutation testing) and a significant increase in positive overshoot (*P* < 0.01, paired *t*-test (Bogacz et al., 2010)] (Figure 6C). Notably, the HRFs in pre-frontal cortex of this minority group exhibited an initial “positive” dip [*P* < 0.01, paired *t*-test (Bogacz et al., 2010)] followed by milder sustained suppression [*P* < 0.01, paired *t*-test (Bogacz et al., 2010)], suggesting an interaction between suppression of mind wandering and enhancement of top-down executive control for the inhibition of task-irrelevant stimuli (Knight et al., 1999; Lee and D'Esposito, 2012).

For completeness, the above HRF plots were also generated for more specific ROIs (V1, V2, V3, V4, MT, pre-motor cortex, inferior parietal lobule PGp, and superior parietal lobule 7P (Supplementary Figures 1, 2)–as defined by the Julich histological atlas in FSL (Eickhoff et al., 2005). Notably, individual early visual areas including MT were well-characterized by the aggregated PVC results (Figure 6). For the parietal regions, no significant difference was found ~6 post stimulus. However, ~2 s post stimulus, these regions followed the results of the aggregated PVC results. No significant difference (Ac vs. SpAc emphasis) was found in the HRFs of premotor cortex.

## Discussion

Recent studies suggest that time pressure results in reduced decision making accuracy [i.e., the speed-accuracy tradeoff (SAT) (Wickelgren, 1977; Forstmann et al., 2008; van Veen et al., 2008; Bogacz et al., 2010)]. The behavioral mechanism behind SAT is thought to be a lowering of response threshold bounds (Forstmann et al., 2008; Bogacz et al., 2010) with no involvement of primary sensory cortices (e.g., PVC) (Forstmann et al., 2008; van Veen et al., 2008; Bogacz et al., 2010) but see Ho et al. (2012). Our results show that SpAc emphasis can improve both speed and accuracy under difficult conditions by increasing drift rate [i.e., perceptual stimulus discriminability (Ratcliff, 1978, 2002; Liu and Watanabe, 2011)]. While these results may be surprising from the perspective of traditional SAT theory, they are consistent with early literature on the interaction between attention, task difficulty, and behavioral/neuronal performance. For example, Spitzer et al. (1988) found that increasing task difficulty enhanced attention and thus behavioral performance as well as strength and selectivity of neural responses from cortical area V4 in rhesus monkeys. Similarly, Rees et al. (1997) found that increasing perceptual load (i.e., task difficulty) in a linguistic task saturated use of attentional resources on task relevant stimuli resulting in improved suppression of task irrelevant motion stimuli by human cortical area V5. Interestingly, similar improvements in both speed and accuracy have been connected to perceptual learning (Liu and Watanabe, 2011) suggesting that SpAc emphasis as a form of time pressure [for the form with speed only emphasis see Forstmann et al. (2008)], may be used to enhance learning. Further investigation could include studies to determine the optimal amount and form of time pressure to achieve best behavioral performance.

Using highly accelerated simultaneous multi-slice fMRI to measure subjects' sub-second whole brain BOLD activity, we found that the neural correlates of this enhancement in stimulus discriminability stems in part from modulation of the initial dip in PVC together with sustained suppression of mPFC. Specifically and importantly, two classes of physiological responses were observed (Figure 6). The majority group of subjects exhibited an enhancement of initial dip amplitude in PVC under SpAc emphasis, the size of which was correlated with the subjects' improvement in drift rate (Figure 6A)—suggesting a continuum of metabolic overdrive states across subjects. Such a quick and early increase in CMRO_2_ would be consistent with prior work on visual attention where brief recruitment of additional attention resources is required for fast, accurate processing of the visual stimulus (Mishra et al., 2011). Other studies have described this phenomenon perceptually as a slowing down of time (Hicks et al., 1977; Coull et al., 2004), which in the context of this experiment would explain how our subjects are able to improve accuracy with less physical time.

In contrast, for the minority group of high performing subjects, initial dip amplitude in PVC was already strong under Ac emphasis (i.e., without time pressure) and actually decreased with SpAc emphasis (Figure 6C). Together with the increase in positive overshoot response under SpAc emphasis, these subjects appear to shift their physiological response to a different, possibly higher, overdrive mode. While we cannot rule out the potential physiological confounds inherent in, for example, time pressure induced stress in this minority group of subjects (Staal, 2004; Muehlhan et al., 2013), their response is remarkably consistent with blockage of adenosine receptors and subsequent quickening of the CBF response [e.g., as has been previously hypothesized in studies of the vasoconstrictive, neurostimulant drug: caffeine; which causes a similar reduction of the initial dip and amplification of the positive overshoot (Behzadi and Liu, 2006)]. Given that adenosine is a homeostatic regulatory factor serving to match the rate of neural energy consumption to the rate of substrate supply [e.g., locally increases CBF and reduces CMRO-_2_ in the presence of strong/persistent CMRO_2_ (Fredholm et al., 1999)], blockage of adenosine receptors or removal of adenosine from extracellular space could enable this additional mode of metabolic overdrive of neural activity and facilitate overcoming of SAT in these subjects. An endogenous mechanism for this has been shown to be regulation of adenosine kinase activity by astrocytes, allowing for control of extracellular adenosine concentration in a neuronal activity-dependent manner (Diogenes et al., 2014).

The possibility of adenosine playing a role in our study could also explain the two classes of BOLD responses we observed. Prior studies have shown that the level of expression of adenosine receptors depended strongly on a subjects' environment/diet (Johansson et al., 1993) with profound impacts on the BOLD response (Laurienti et al., 2002). For example, in the study of Laurienti et al. (2002), not only was it found that subjects with likely higher expression of adenosine receptors (due to high levels dietary caffeine) had stronger BOLD responses but it was also found that these subjects further amplified their BOLD responses post-caffeine ingestion. However, there are many other factors that contribute to variability in the BOLD response across subjects (Aguirre et al., 1998; Handwerker et al., 2004; Buxton, 2013). Future studies on the effect of time pressure on the BOLD responses controlling for additional factors including physiological stress response (e.g., heart rate, blood pressure, cortisol levels) and frequency of past exposure to stressful or time-pressured situations, would help shed light on the two classes of physiological responses found in our study.

Also notable are the initial positive dip and reduction in sustained suppression in mPFC of the minority group of subjects under SpAc emphasis. While we are not aware of any prior reports of an initial positive dip, its rapid ~2 s peak suggest a biophysical mechanism analogous to that of the initial negative dip: a *decrease* in CMRO_2_ and/or CBV prior to a vascular response of *decreased* CBF. This would be consistent with a rapid and early suppression of mind wandering (Gusnard et al., 2001; Raichle et al., 2001; Mason et al., 2007; Singh and Fawcett, 2008). As for the reduction in sustained suppression, one possible explanation is that, for optimal behavioral performance, these subjects not only suppress mind wandering but also enhance top-down executive control of processing task-relevant stimuli and inhibition of task-irrelevant stimuli, which has been shown to increase BOLD activity in mPFC (Knight et al., 1999; Lee and D'Esposito, 2012).

Prior studies found the anterior-striatum and the pre-supplementary motor area (pre-SMA) to be involved in time-pressured decision making (Forstmann et al., 2008; Bogacz et al., 2010). It was found that BOLD activity in these areas was stronger in subjects that were less cautious while under time pressure. However, we did not find significant effects on the BOLD signal in these areas. This is likely due to the difference between speed (Sp) emphasis and SpAc emphasis. While subjects in Forstmann et al. (2008) and Bogacz et al. (2010) were instructed to regard accuracy less importantly during Sp conditions, our subjects were instructed to treat accuracy equally important under both task conditions (Ac and SpAc). Furthermore, in contrast to our study, Forstmann et al. (2008) and Bogacz et al. (2010) did not find time-pressure related BOLD activity in PVC. Although this may be due to the distinction between Sp and SpAc emphasis it may also be due in part to the elusive nature of the initial dip (Ances, 2004; Vanzetta and Grinvald, 2008; Hu and Yacoub, 2012) compounded by their relatively slow fMRI sampling rate and thus focus on the positive overshoot BOLD response. By using the higher sampling rates possible with simultaneous multi-slice EPI (Feinberg et al., 2010; Moeller et al., 2010; Setsompop et al., 2012) and clustering subjects with respect to their behavioral and physiological responses, we were able to reliably detect changes in PVC initial dip that was not evident in the positive overshoot (Figure 6). This dissociation between the initial dip and positive overshoot provides evidence that the BOLD signal contains much more information at higher temporal frequencies and lower latencies than previously believed (Nishimoto et al., 2011) and further demonstrates the advantage of sub-second whole brain fMRI. Furthermore, the initial dip/positive overshoot dissociation, in conjunction with the strong relationship between initial dip strength and drift rate improvement, suggests that the initial dip can serve as an independent biomarker and opens the door to novel cognitive neuroscience experiments where the traditional positive overshoot maybe less sensitive or less spatially specific to underlying neural activity of interest.

## Data Availability Statement

The raw data supporting the conclusions of this article will be made available by the authors, without undue reservation.

## Ethics Statement

The studies involving human participants were reviewed and approved by Committee for the Protection for Human Subjects at the University of California, Berkeley. The patients/participants provided their written informed consent to participate in this study.

## Author Contributions

AV and DF designed the experiment and wrote the paper. AV collected the data and analyzed the data. Both authors contributed to the article and approved the submitted version.

## Conflict of Interest

DF is employed by Advanced MRI Technologies, LLC that developed MRI pulse sequences used for conducting these experiments. The remaining author declares that the research was conducted in the absence of any commercial or financial relationships that could be construed as a potential conflict of interest.

## Supplementary Material

Supplementary Figure 1 Majority group mean HRFs in specific ROIs. Format is the same as Figure 6 but for the following ROIs: V1, V2, V3, V4, MT, premotor cortex, inferior parietal lobule PGp, and superior parietal lobule 7P–as defined by the Julich histological atlas.

Supplementary Figure 2 Minority group mean HRFs in specific ROIs. Format is the same as Figure 6 but for the following ROIs: V1, V2, V3, V4, MT, premotor cortex, inferior parietal lobule PGp, and superior parietal lobule 7P–as defined by the Julich histological atlas.

Supplementary Figure 3 Stimulus matrix design. **(A)** Stimulus matrix used to account for stimulus duration. The full matrix dimensions with three runs are 2,790 timepoints × 288 parameters. Only 400 time points are shown for visibility. The matrix is the concatenation of the stimulus convolution matrix for each of the 6 event types (task difficulties). The stimulus convolution matrix for a given event type consists of a shifted binary sequences indicating event occurrences. There are 48 shifts (16 s), one for each time point in the HRF estimate. Stimulus duration, determined by subjects' response time, is accounted for by replicating the shifted binary sequence a corresponding number of timepoints. Durations less than the TR are scaled proportionally. The inset (upper-left) is an enlarged view of the parameters for the first two event types. **(B)** Stimulus matrix without accounting for stimulus duration FIR model in Kay et al. (2008).
