# Supplementary figures and images for: The Role of Cerebral Metabolism in Improving Time Pressured Decisions
Source: Front Psychol. 2021 Jul 20;12:690198. doi: 10.3389/fpsyg.2021.690198 (PMC8329240; doi:10.3389/fpsyg.2021.690198)

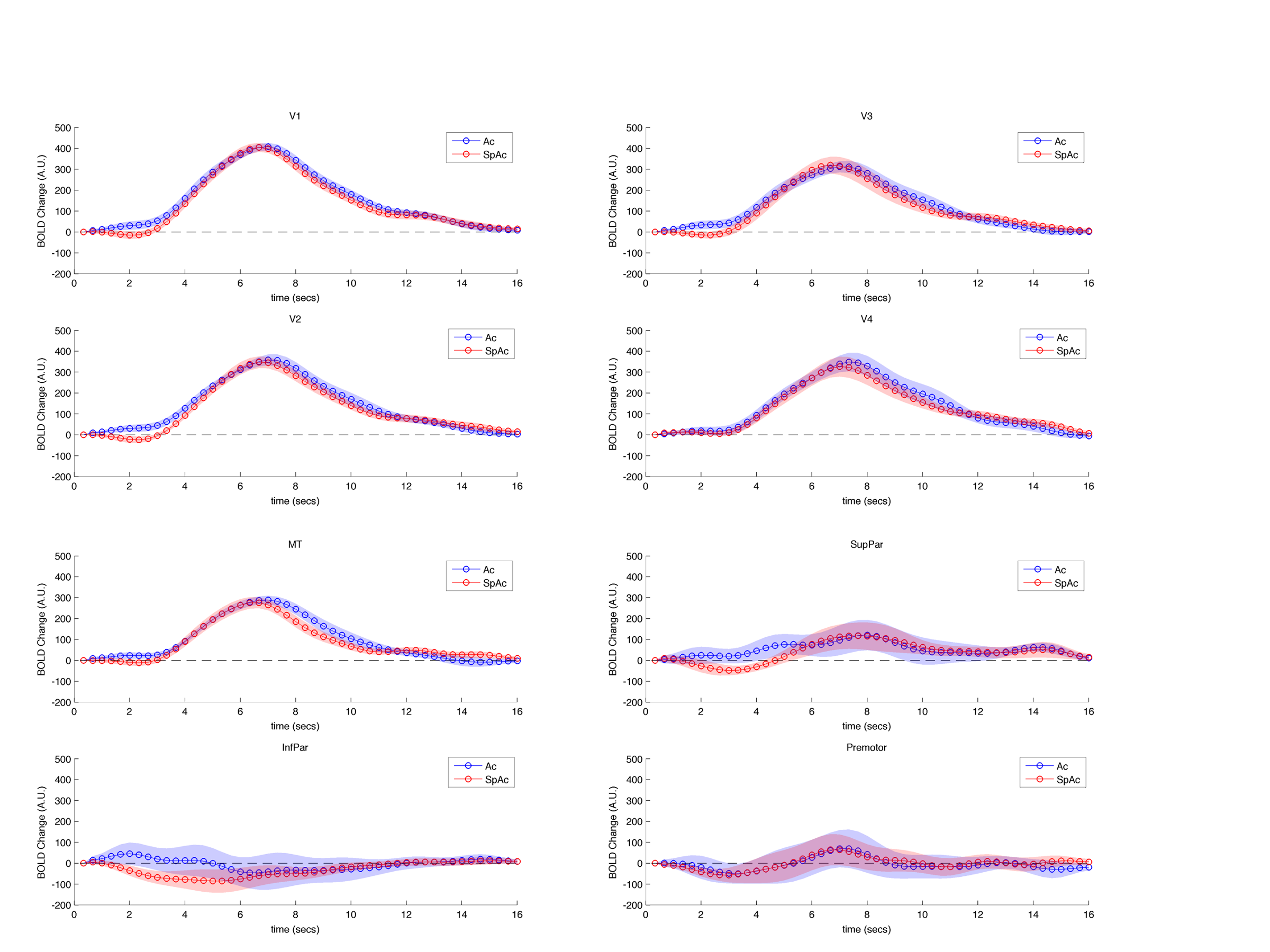

Supplement: Supplementary Figure 1 — Majority group mean HRFs in specific ROIs. Format is the same as Figure 6 but for the following ROIs: V1, V2, V3, V4, MT, premotor cortex, inferior parietal lobule PGp, and superior parietal lobule 7P–as defined by the Julich histological atlas. [file Image_1.TIFF]

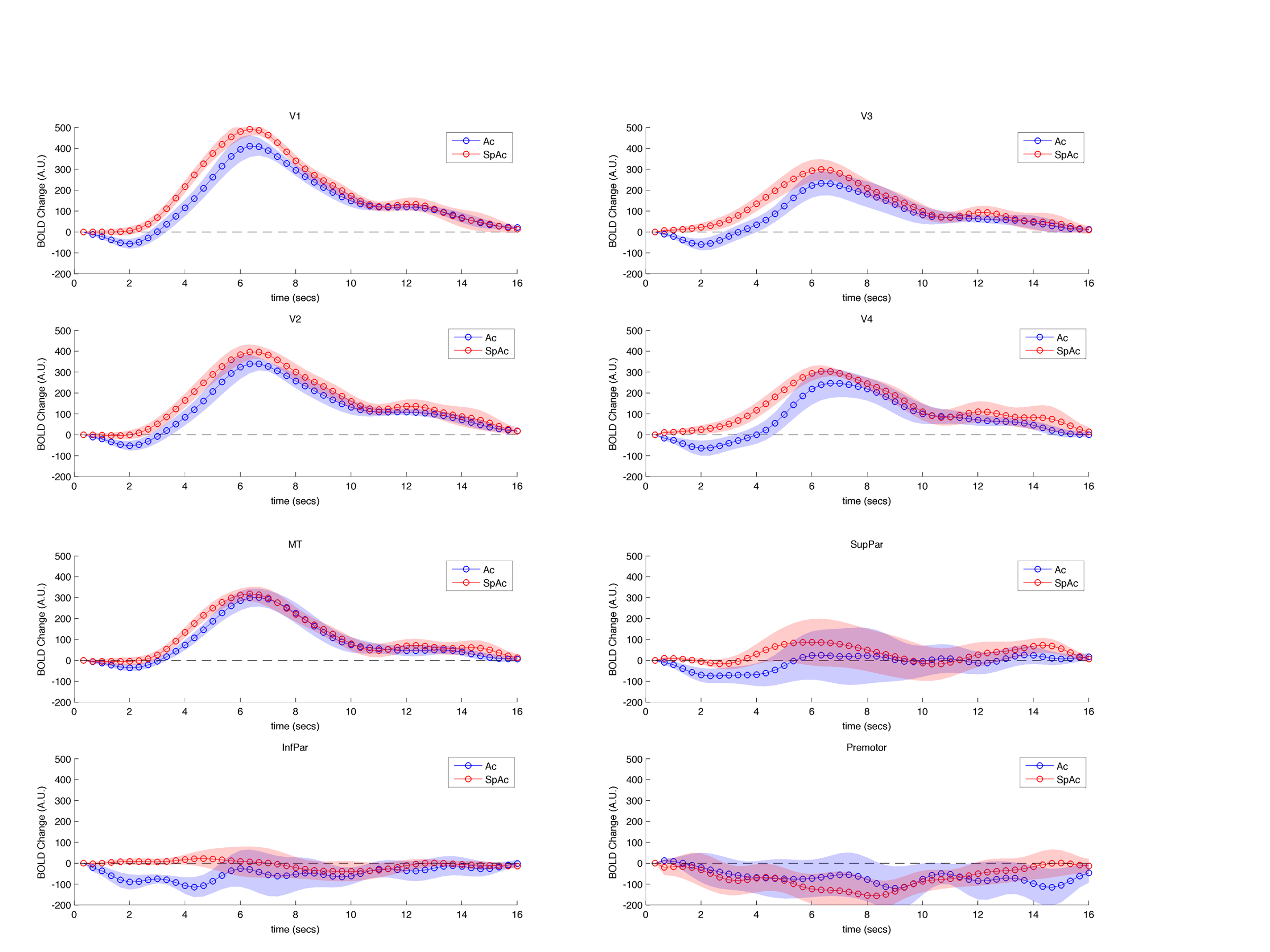

Supplement: Supplementary Figure 2 — Minority group mean HRFs in specific ROIs. Format is the same as Figure 6 but for the following ROIs: V1, V2, V3, V4, MT, premotor cortex, inferior parietal lobule PGp, and superior parietal lobule 7P–as defined by the Julich histological atlas. [file Image_2.TIFF]

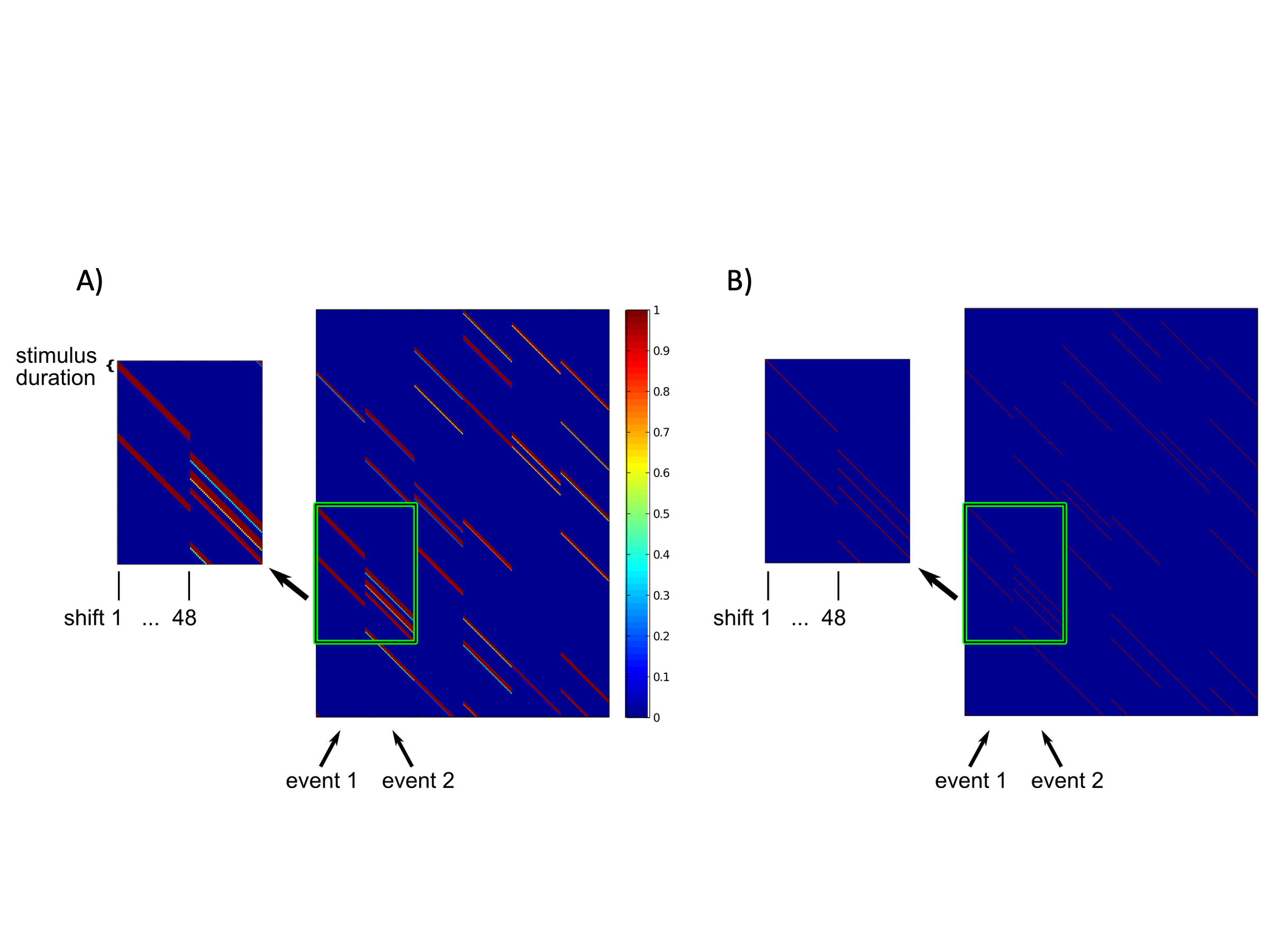

Supplement: Supplementary Figure 3 — Stimulus matrix design. (A) Stimulus matrix used to account for stimulus duration. The full matrix dimensions with three runs are 2,790 timepoints × 288 parameters. Only 400 time points are shown for visibility. The matrix is the concatenation of the stimulus convolution matrix for each of the 6 event types (task difficulties). The stimulus convolution matrix for a given event type consists of a shifted binary sequences indicating event occurrences. There are 48 shifts (16 s), one for each time point in the HRF estimate. Stimulus duration, determined by subjects' response time, is accounted for by replicating the shifted binary sequence a corresponding number of timepoints. Durations less than the TR are scaled proportionally. The inset (upper-left) is an enlarged view of the parameters for the first two event types. (B) Stimulus matrix without accounting for stimulus duration FIR model in Kay et al. (2008). [file Image_3.TIFF]
